# IL-12 Gene Electrotransfer Triggers a Change in Immune Response within Mouse Tumors

**DOI:** 10.3390/cancers10120498

**Published:** 2018-12-08

**Authors:** Guilan Shi, Chelsea Edelblute, Sezgi Arpag, Cathryn Lundberg, Richard Heller

**Affiliations:** 1 Frank Reidy Research Center for Bioelectrics, Old Dominion Unviersity, Norfolk, VA 23508, USA; gshi@odu.edu (G.S.); cedelblu@odu.edu (C.E.); sarpagmc@odu.edu (S.A.); clundber@odu.edu (C.L.); 2 School of Medical Diagnostics and Translational Science, Old Dominion University, Norfolk, VA 23508, USA

**Keywords:** immunotherapy, gene therapy, melanoma, IL-12, electrotransfer

## Abstract

Metastatic melanoma is an aggressive skin cancer with a relatively low survival rate. Immune-based therapies have shown promise in the treatment of melanoma, but overall complete response rates are still low. Previous studies have demonstrated the potential of plasmid IL-12 (pIL-12) delivered by gene electrotransfer (GET) to be an effective immunotherapy for melanoma. However, events occurring in the tumor microenvironment following delivery have not been delineated. Therefore, utilizing a B16F10 mouse melanoma model, we evaluated changes in the tumor microenvironment following delivery of pIL-12 using different GET parameters or injection of plasmid alone. The results revealed a unique immune cell composition after intratumoral injection of pIL-12 GET. The number of immune memory cells was markedly increased in pIL-12 GET melanoma groups compared to control group. This was validated using flow cytometry to analyze peripheral blood mononuclear cells as well as delineating immune cell content using immunohistochemistry. Significant differences in multiple cell types were observed, including CD8^+^ T cells, regulatory T cells and myeloid cells, which were induced to mount a CD8^+^PD1^−^ T cells immune response. Taken together, these findings suggest a basic understanding of the sequence of immune activity following pIL-12 GET and also illuminates that adjuvant immunotherapy can have a positive influence on the host immune response to cancer.

## 1. Introduction

Effective treatment options for melanoma are significantly limited, especially for metastatic melanoma and recurrent disease. Surgery and radiotherapy mainly provide palliation, and chemotherapy has failed to show any consistent survival benefit [1,2]. To effectively treat metastatic disease with immunotherapy, it is important to induce a systemic immune response and/or induce memory T cells that can specifically target the primary tumor or recurrences [3]. Interleukin 12 (IL-12) is an ideal candidate for tumor immunotherapy, due to its ability to activate both innate (NK cells) and adaptive (cytotoxic T lymphocytes) responses and to potently stimulate the production of IFN-γ leading to a potentially effective anticancer defense [4,5]. However, despite encouraging results in animal models, very modest antitumor effects of IL-12 were achieved in early clinical trials, often accompanied by unacceptable levels of adverse events, which markedly dampened hopes of the successful use of this cytokine in cancer patients [6,7].

An alternative strategy to enhance an antitumor immune response is to deliver the cytokine gene directly into tumors. Previous studies have found that IL-12 plasmid delivered by in vivo electroporation (pIL-12 GET) generated successful treatment of established subcutaneous melanoma tumors with minimal to no toxicity in both preclinical and clinical studies [3,8,9,10]. Local expression of IL-12 led to recruitment of immune cells infiltrating the tumor microenvironment, which have been directly associated with favorable clinical outcomes, including disease-free survival, and overall survival in various solid tumors, including melanoma [11,12].

Tumor-infiltrating lymphocytes (TILs) are a heterogeneous group comprised of not only effector T cells, but also of tolerogenic or T regulatory (Treg) cells, functionally exhausted T cells, myeloid-derived suppressor cells (MDSCs), and other immune cell types. Thus, mapping the immune compartment of B16F10 melanoma tumors could allow a better understanding of the immunologic circuits active after pIL-12 GET in this malignancy.

The present study was designed to evaluate changes in the makeup of peripheral blood mononuclear cells (PBMCs) and immune cells within the tumor microenvironment following pIL-12 GET. Peripheral blood analysis was included to provide insights about the immune response induced following pIL-12 GET, furthermore, peripheral blood analyses may provide a systemic view of the immune response and can be repeated at several time points. Local antitumor immunity was analyzed by the enumeration of tumor-infiltrating lymphocytes. The results demonstrated that pIL-12 GET results in decreased levels of PD1 expression on CD4^+^ and CD8^+^ T cells, percentage of Treg cells in the tumor and increased infiltration of CD8^+^PD1^−^ T cells leading to better control of tumor growth, strongly suggesting that therapy with pIL-12 GET has the potential to improve outcomes.

## 2. Results

### 2.1. Tumor Regression and Protection Against Rechallenge by pIL-12 GET

**Figure 1:**
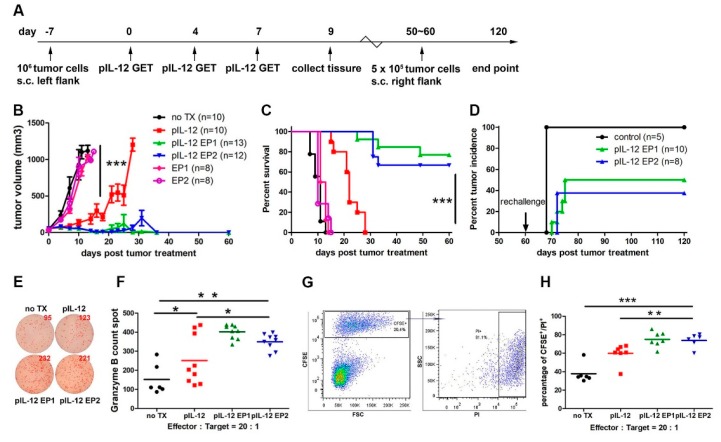
Antitumor efficacy in B16F10 tumor-bearing mice treated with IL-12 plasmid delivered by in vivo electroporation (pIL-12 GET) and protective immunity against tumor rechallenge. (**A**) Experimental scheme. On day-7, C57BL/6 mice were inoculated with B16F10 cells (1 × 10^6^/50 μL, s.c in the left flank.). Tumor-bearing C57BL/6 mice were treated with pIL-12 GET on days 0, 4 and 7. Spleen and tumor tissues were collected on day 9. On day 60, long term surviving tumor-free mice were rechallenged by injection of B16F10 cells at half dose (5 × 10^5^/50 μL). The end point time of experiment was day 120. (**B**) Tumor volume was monitored and recorded every 2–3 days until the tumor volume reached the end point. (**C**) Overall survival was determined throughout a 50-day time course. (**D**) Percentage of tumor incidence after rechallenge. (**E**, **F**) On day 9, splenocytes (1 × 10^5^) from B16F10 tumor-bearing mice were incubated with B16F10 target cells for 48 h in 96-well plates in triplicate (200 μL/well) at ratio of 20:1. Granzyme B spots counted in enzyme-linked immunospot (ELISPOT). (**G**,**H**) Splenocytes (1 × 10^6^) from B16F10 tumor-bearing mice were incubated with carboxyfluorscein succinimidyl ester (CFSE) stained-B16F10 target cells in u-bottom tube in triplicate at ratio of 20:1 for 4 h. The cytotoxic activity was measured by flow cytometric analysis comparing CFSE^+^PI^+^cells (killed targets) with CFSE^+^PI^-^cells killing. Pooled data from two independent experiments are shown. Each value represents the mean +/− SEM of the group (animals in each group, *n* = 8–13). One-way ANOVA, *p* * < 0.05, *p* ** < 0.01, *p* ***< 0.001.

Motivated by the synergy observed between expression kinetics and expression levels of IL-12 related to the applied electric pulse parameters in our previous study [10], we explored two different GET treatments, pIL-12 EP1 and pIL-12 EP2. An intratumoral injection of pIL-12 followed by electroporation pulsing sequence 1 (EP1—six 1300 V/cm, 100 μs pulses with an array of 6 penetrating electrodes) or electroporation pulsing sequence 2 (EP2—ten 600 V/cm, 5 ms pulses with a non-penetrating caliper electrodes) was performed 3 times on days 0, 4 and 7 (Figure 1A). pIL-12 GET induced a robust tumor regression (Figure 1B) compared with no treatment (TX), pIL-12 injection only, EP1 no IL-12 and EP2 no IL-12 groups. A significant difference in reduction of tumor volume was observed with pIL-12 GET (*p* < 0.001). Durable cures of around 80% in pIL-12 EP1 and 65% in pIL-12 EP2 of mice bearing B16F10 melanoma tumors (Figure 1C), while pIL-12 injection only elicited weaker therapeutic response (Figure 1B, C). 50–60 days after cessation of therapy, tumor free mice were rechallenged with an injection of 5 × 10^5^ B16F10 cells into the opposite flank. Around 50% of the pIL-12 GET mice remained tumor free after the rechallenge, suggesting the induction of effective immunological memory (Figure 1D). Importantly, despite high rates of response, pIL-12 GET therapy was associated with minimal systemic toxicity, as mice did not show weight loss (Figure S1). In previous experiments, we have not observed tumor recurrence in mice that were tumor-free at 120 days, even after regular observation for more than a half year, and therefore in the present study, mice that were tumor-free at 120 days were deemed to have mounted a long-term response and were euthanized [10]. 

To better understand the response, anti-tumor cell activity present following three pIL-12 GET treatments was analyzed. Spleens were collected on Day 9 and splenocytes isolated. Splenocytes were co-cultured with B16F10 melanoma cells for 2 days and then evaluated by ELISPOT. Since the tumor volume and percentage of survival were similar among no TX, EP1 and EP2 group, the next analysis was performed on no TX, pIL-12, pIL-12 EP1 and pIL-12 EP2 groups. Splenocytes isolated from mice treated with pIL-12 GET released more Granzyme B compared to no TX and pIL-12 injection only groups (Figure 1F,G). Similarly, in a flow cytometry killing assay, splenocytes from pIL-12 EP1 and pIL-12 EP2 groups achieved higher cytotoxic activity to kill B16F10 melanoma target cells (Figure 1H,I). These results suggest that the enhanced antitumor efficacy of pIL-12 GET treatment was associated with the reduced-tumor volume and prolonged-survival. Furthermore, pIL-12 GET induced an immune memory response that protected against rechallenge.

### 2.2. pIL-12 GET Therapy Impacts Tumor Immune Infiltration

**Figure 2:**
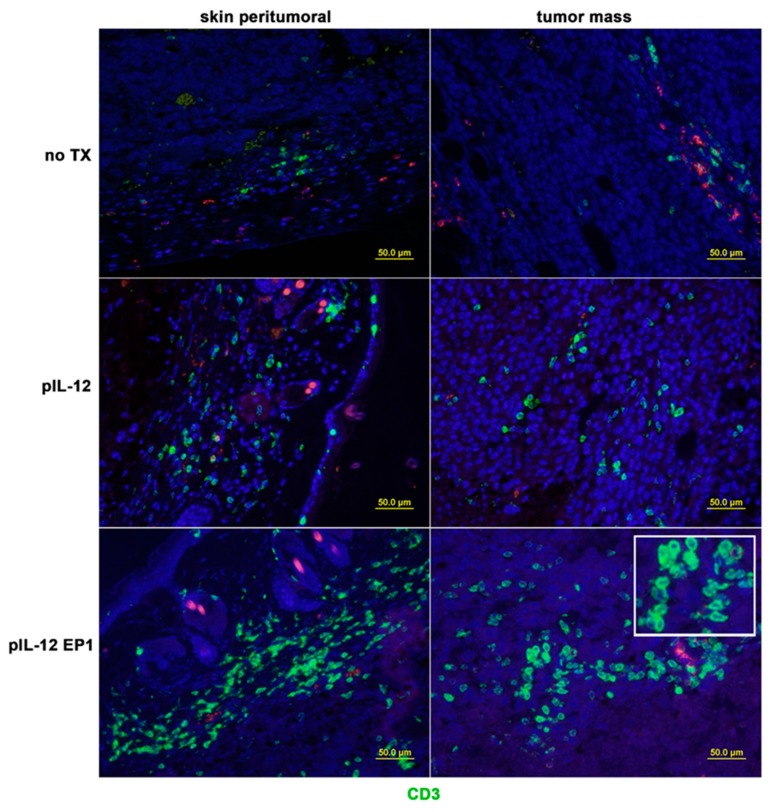

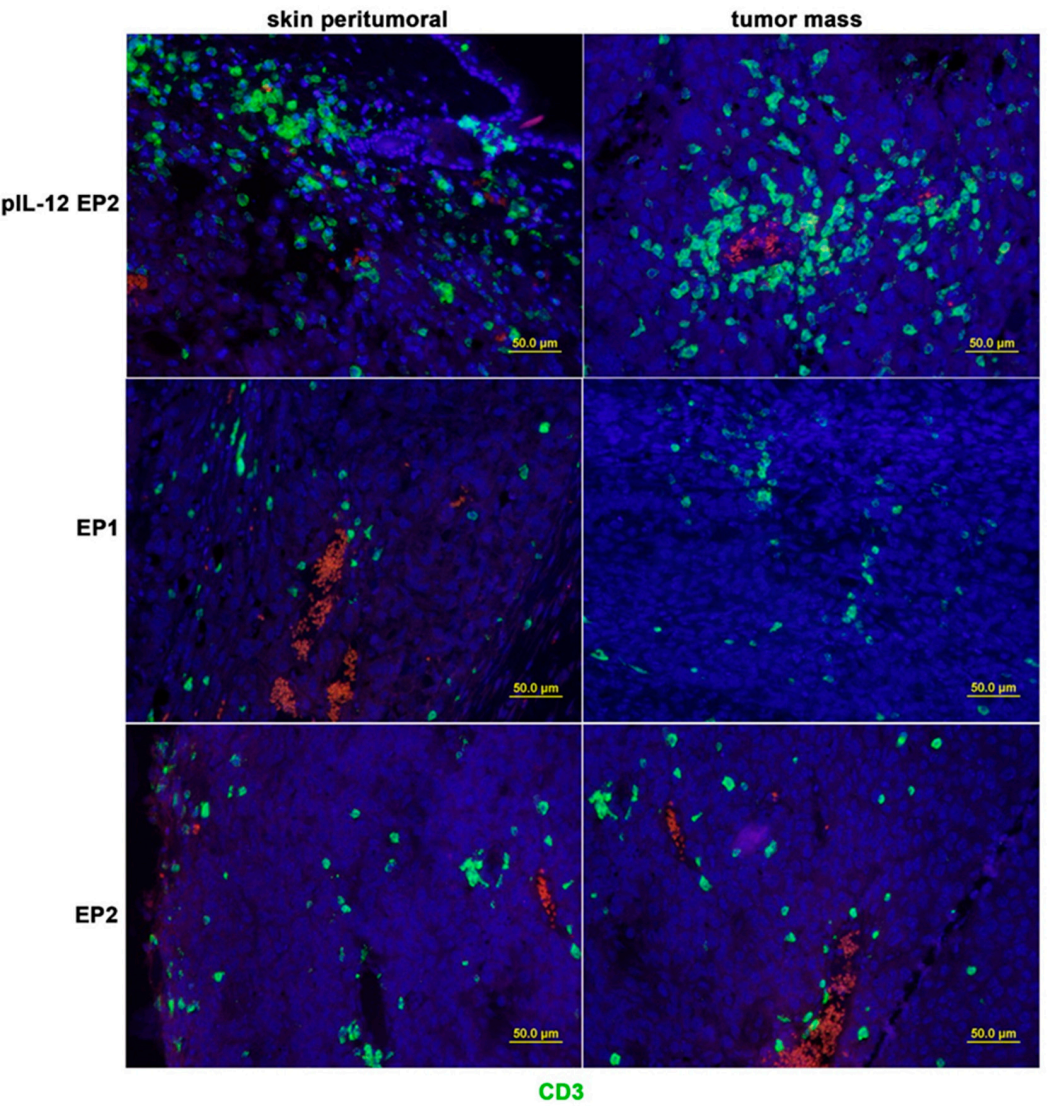
pIL-12 GET impacts tumor immune infiltration location. Tumors were collected at day 9 for histological analysis. Paraffin sections of tumor tissue were stained with anti-CD3 (green, Opal 520). Opal 570 (red color) was used to confirm the non-specific staining of anti-CD3. Results are given as the location of CD3^+^ cells from the different groups. Three mice per group. Visualized by optical microscopy (×200). Red: Opal 570; Green: Opal 520; Blue: 4’,6-diamidino-2-phenylindole (DAPI).

The majority of immunotherapeutic approaches are based on the ability of the adaptive immune system to infiltrate tumors and elicit an anti-tumor response. To understand the cellular mechanism underlying the observed therapeutic effect of pIL-12 GET therapy, we further analyzed the immune response in the B16F10 melanoma model, which represents an immunosuppressive tumor microenvironment with low expression of MHC class I (MHC-I) molecules and high PDL1 expression (Figure S2). The loss of MHC-I expression on tumor cells is an immune escape strategy aimed to avoid T-cell recognition which is commonly found in malignant cells. In addition, one of the protective mechanisms utilized by melanoma cells is through overexpression of PD-L1; as a result, it circumvents the generation of an immune response to the tumor. We observed that most of the immune cells formed aggregates and resided outside the tumor mass although they were in close proximity to tumors, and blood vessels. However, after pIL-12 GET, there was an increase of lymphocytic infiltrate into the tumor mass (Figure 2).

**Figure 3:**
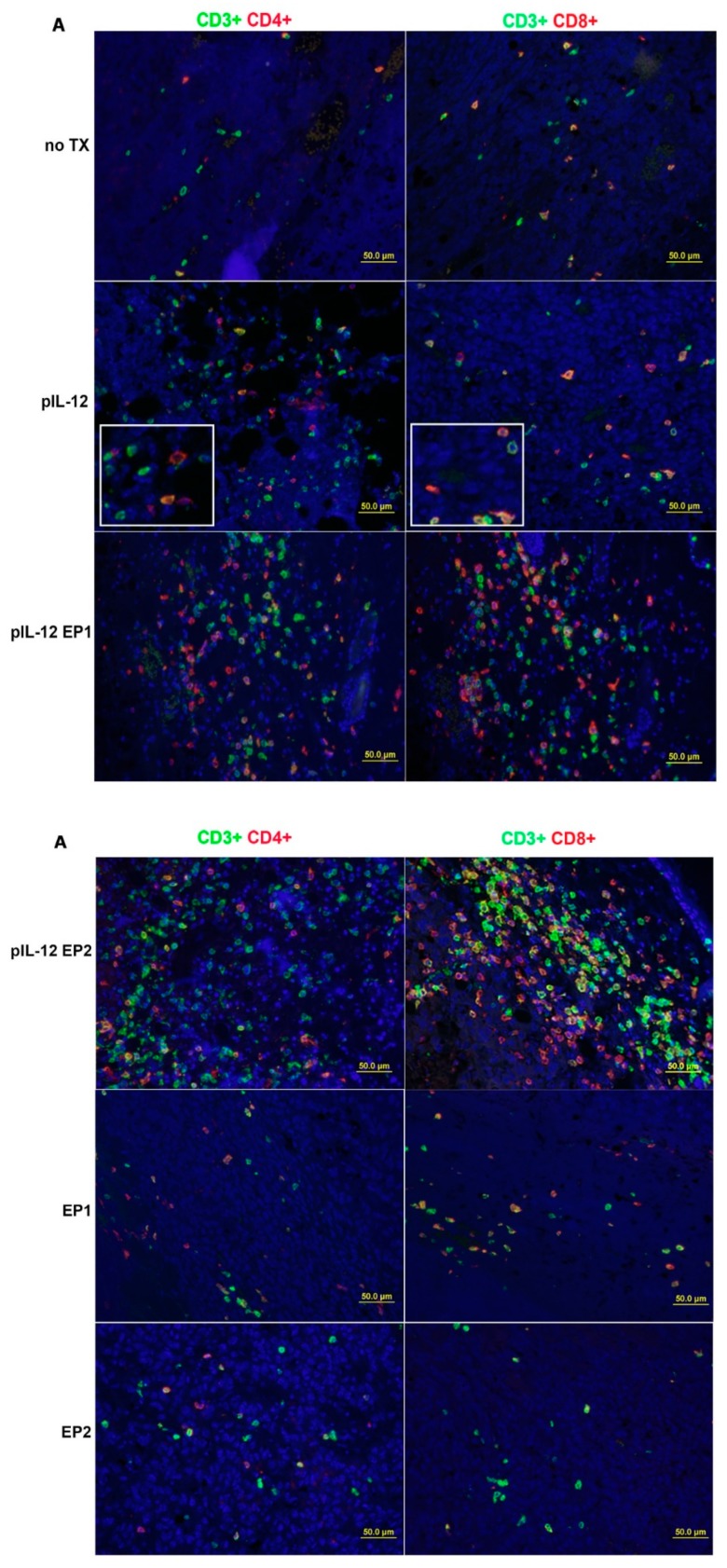

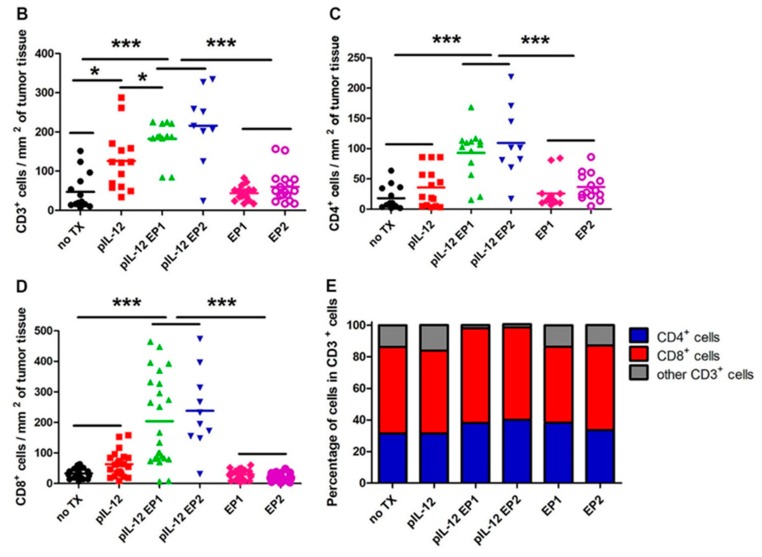
pIL-12 GET enhances tumor infiltrating lymphocyte (TILs) density in the Tumor Microenvironment. (**A**) Tumors were collected on day 9 for histological analysis. Paraffin sections of tumor tissue were stained with anti-CD3, anti-CD4 and anti-CD8. Visualized by optical microscope (×200). (**B**–**D**) Representative immunohistochemical analysis of pIL-12 GET. The density of TILs was quantified in 10–20 random fields (number of total cells > 200). Horizontal bars indicate mean values. The data presented are representative of two independent experiments. (**B**) CD3^+^ density. (**C, D**) IHC data represented as the mean density of CD4^+^ and CD8^+^. (**E**) The percentage of CD4^+^ and CD8^+^ cells among total CD3^+^ cells. Each value represents the mean +/− SEM of the group. Pooled data from two independent experiments are shown. One-way ANOVA, *p* * < 0.05, *p* ***< 0.001.

**Figure 4:**
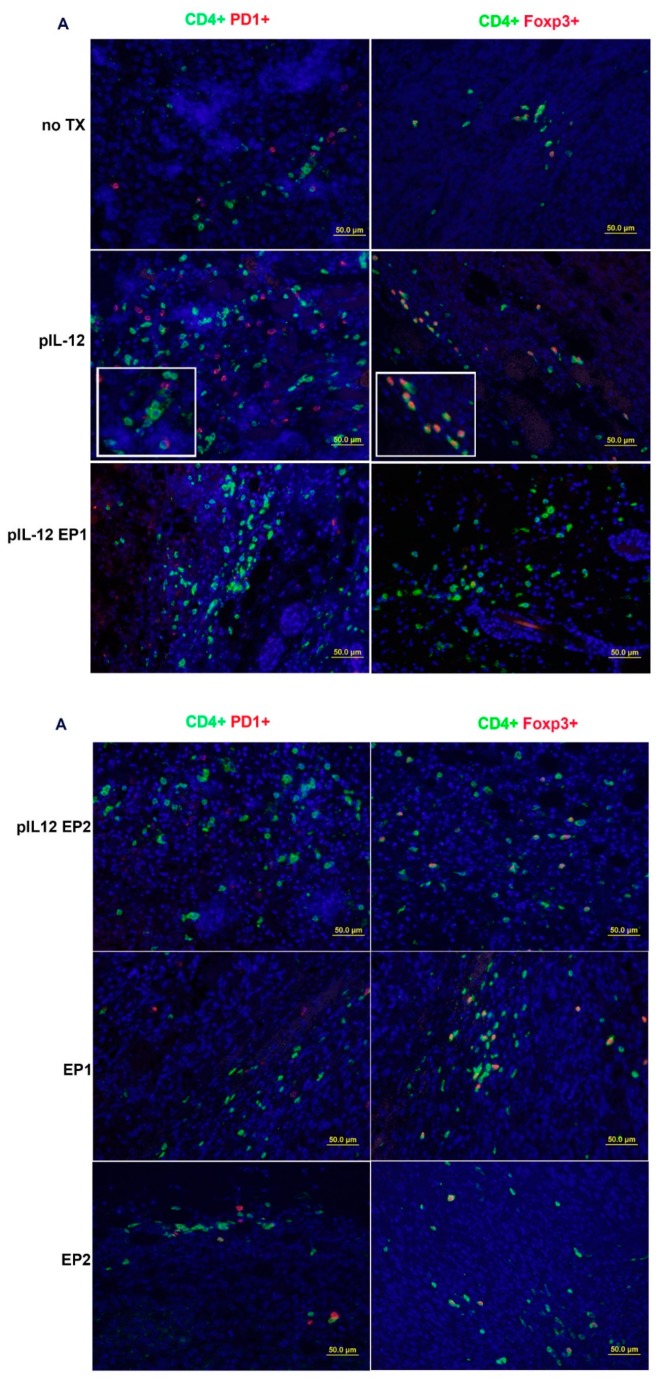

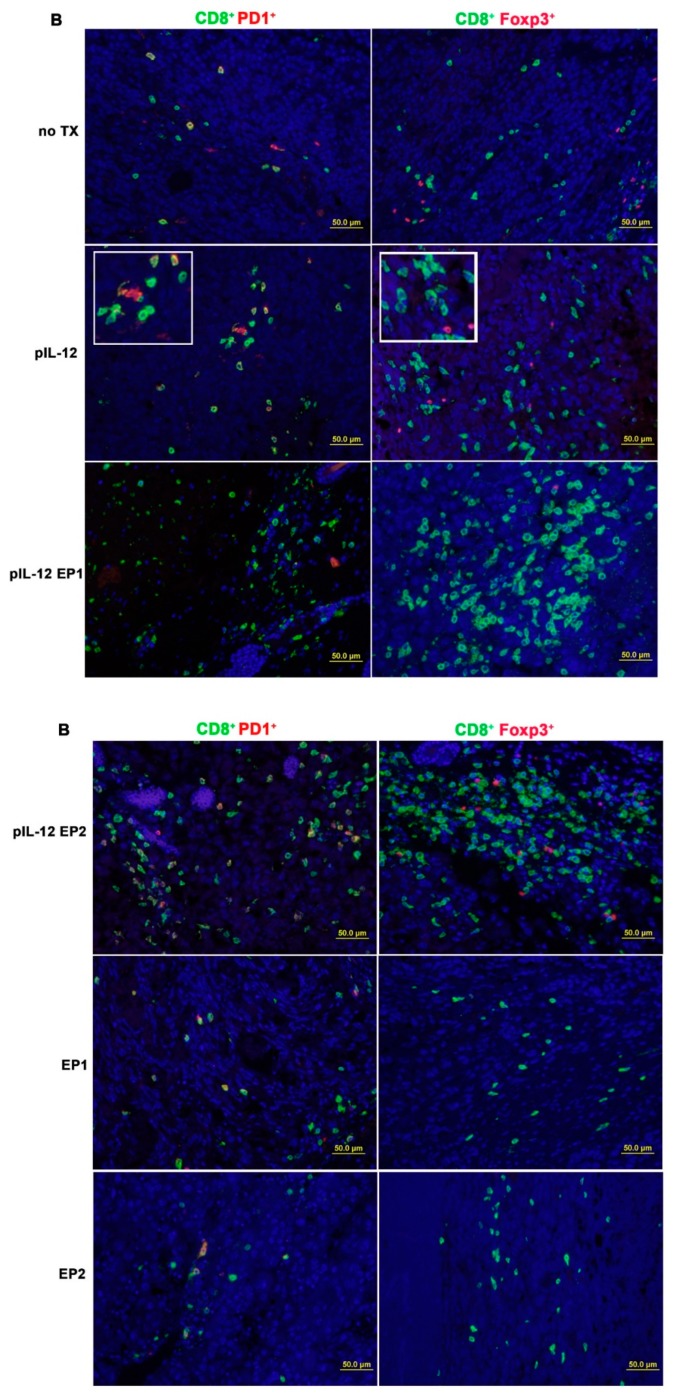

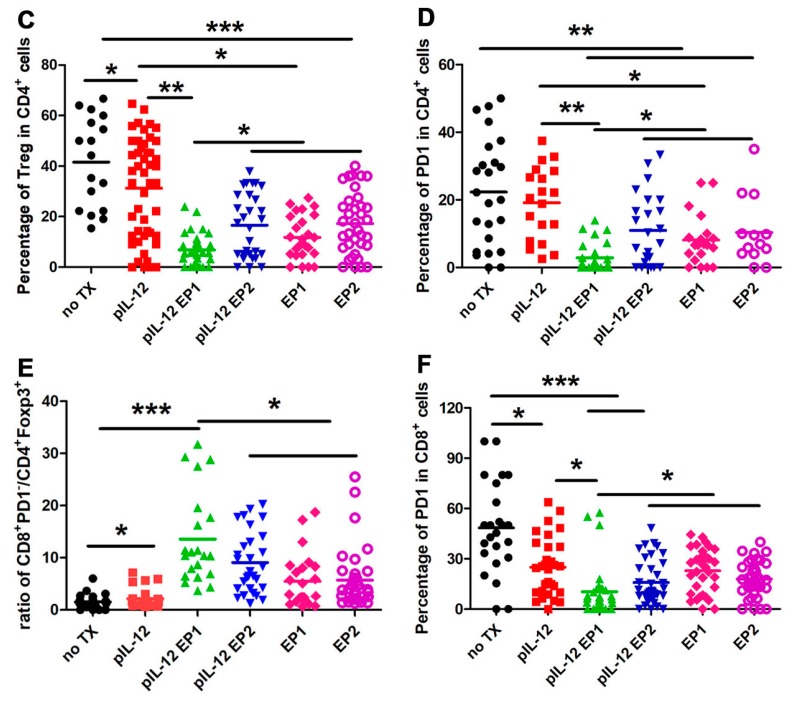
Changes in the Tumor Microenvironment following pmIL-12 GET. Tumors were collected at day 9 for histological analysis. (**A**) Paraffin sections of tumor tissue were stained with anti-CD4, anti-Foxp3 and anti-PD1. (**B**) Paraffin sections of tumor tissue were stained with anti-CD4, anti-Foxp3 and anti-PD1. Visualized by optical microscope (×200). (**C**, **D**) IHC data represented as the mean percentage of Treg cells in CD4^+^ and exhausted CD4^+^ PD1^+^ cells in CD4^+^ cells in four different groups. (**E**, **F**) The ratio of CD8^+^ T cells to CD4^+^Foxp3^+^ Treg cells. The data presented are representative of two independent experiments. Each value represents the mean +/− SEM of the group. The percentage of TIL was quantified in 10–20 random fields (number of total cells > 200). Horizontal bars indicate mean values. One-way ANOVA, *p* * < 0.05, *p* ** < 0.01, *p* ***< 0.001.

The distribution of TILs changed following pIL-12 GET treatment resulting in an expansion of CD3^+^ T cells infiltrating in the tumor environment, which involved CD4^+^ and CD8^+^ T cells (Figure 3A) compared with no TX, EP1, EP2 and pIL-12 injection only groups. An abundance of these cells is typically associated with favorable response to immunotherapy. Quantification of tumor-infiltrating lymphocytes revealed more lymphocytes infiltrated in the tumor microenvironment with effective therapies (Figure 3B–E). There was a noticeable difference comparing pIL-12 GET groups to no TX, EP1, EP2 and pIL-12 injection only groups (*p* < 0.001) in density of CD3^+^, CD4^+^ and CD8^+^ T cells. In comparing the 6 groups, we observed no significant difference in the ratios of CD4^+^/CD8^+^ T cells among groups, but the ratio was reversed Further characterization of the microenvironment evaluated the presence of T regulatory cells (Treg) and the presence of T cells positive for PD1. There was a significant decrease in percentage of PD1 positive cells, both CD4^+^ and CD8^+^ cells, and Treg cells following treatment with pIL-12 GET with EP1 (Figure 4). Consistent with IHC results, evaluation of cell populations by flow cytometry revealed a reduction of Treg cells in CD4^+^ T cells and PD1^+^ in CD4^+^ or CD8^+^ T cells after pIL-12 GET or EP1 and EP2 treatment (Figure S3). 

### 2.3. A Dynamic Immune Response Occurs in Mice with B16F10 Tumors after pIL-12 GET 

We next decided to perform a study of the immune kinetics over the full course of treatment. In B16F10 melanoma tumor-bearing mice, pIL-12 EP1 and pIL-12 EP2 treatment partially controlled disease progression and around 65–80% of mice achieved long-term disease-free survival (Figure 1C). The near equal efficacy in tumor control with pIL12 EP1 as compared to pIL12 EP2. Thus, we pooled pIL-12 EP1 and pIL-12 EP2 groups. Two outcomes were observed, long-term tumor free survival. (termed: complete response, CR), or mice that had early tumor control followed by progressive outgrowth (termed: partial response, PR). To allow for the dynamic assessment of the immune response and to explore the mechanism underlying the pIL-12 GET, peripheral blood mononuclear cells (PBMC) from B16F10 melanoma tumor-bearing mice were evaluated at pre-treatment, 9, 17, 27, 37, 47, 60 days post treatment with flow cytometry.

**Figure 5:**
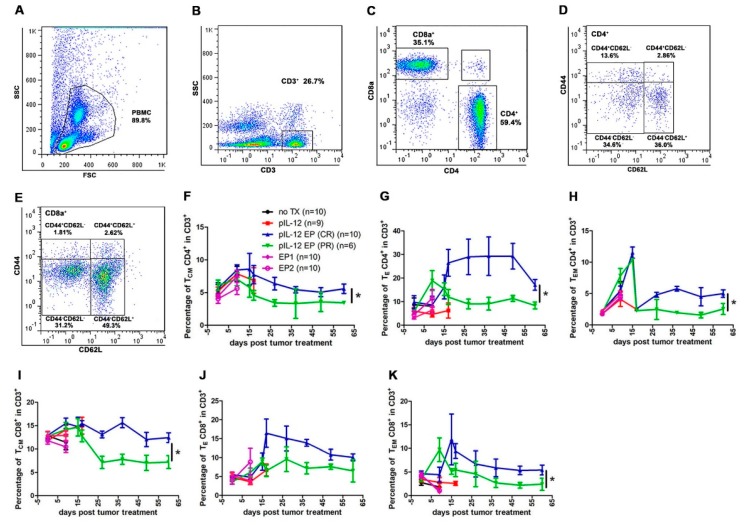
Immunomodulatory effects of pIL-12 GET in B16F10 melanoma model. pIL-12 GET induced an immune response and enhanced percentage of effector CD4^+^, CD8^+^ cells and memory CD4^+^, CD8^+^ cells percentage at different time points. Blood was collected at pre-treatment, and at 9, 17, 27, 37, 49 and 60 days post treatment. Fresh whole blood was used for staining and flow cytometry at each time point. After staining with antibodies, immune cell subsets were detected with flow cytometry. (**A**–**E**) Flow cytometry gating strategy used for defining immune cell subsets. (**F**–**H**) Kinetics of the changes of immune cell subsets in CD4^+^ cells. (**I**–**K**) Kinetics of the changes of immune cells subsets in CD8^+^ cells. The data presented are representative of two independent experiments. Each value represents the mean +/− SEM of the group (animals in each group, *n* = 6–10). One-way ANOVA, *p* * < 0.05.

In mice, CD4^+^ and CD8^+^ T cells can be further categorized into memory and naïve phenotypes based on CD44 and CD62L expression with the CD44^−^CD62L^−^ population considered naïve T cells (T_N_), CD44^+^CD62L^+^ population considered central memory T cells (T_CM_), CD44^+^CD62L^−^ population considered effector memory T cells (T_EM_) and CD44^−^CD62L^−^ (T_E_) population considered effector T cells. It is known that CD4^+^ and CD8^+^ cells differ in their distribution of these subsets in central immune organs, peripheral immune organs and peripheral blood circulation [2,9,13,14,15]. To better understand the mechanism behind the pIL-12 GET induced tumor regression, we evaluated CD4^+^ and CD8^+^ T cell memory phenotype status in peripheral blood at different time points. T_CM_ CD4^+^ and CD8^+^ cells increased around 2-fold in CR mice at all the time points evaluated (Figure 5F,I). T_EM_ CD4^+^ and CD8^+^ populations were significantly increased in CR groups (Figure 5H,K).

**Figure 6:**
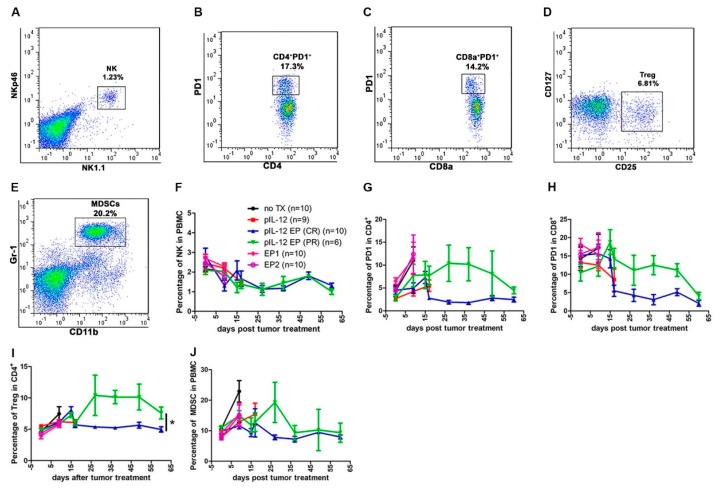
pIL-12 GET decreased CD4^+^PD1^−^, CD8^+^PD1^−^, Treg cells and MDSCs. (**A**–**E**) Representative flow cytometry gating strategy to assess immune cell response to pIL-12 GET. (**F**) Kinetics of the percentage of NK cells. (**G**, **H**) Kinetics of changes of exhausted CD4^+^ and exhausted CD8^+^ cells. (**I**) Kinetics of the percentage of Treg cells. (**J**) Kinetics of the percentage of MDSCs. Pooled data from two independent experiments are shown as mean +/− SEM (animals in each group, *n* = 6–10). One-way ANOVA, *p* * < 0.05.

We observed the proportion of immune cells changing with tumor size at different time points. T_E_ CD4^+^ and CD8^+^ cells were induced in all groups. There was higher percentages in mice achieving a CR compared with mice achieving PR (Figure 5G,J). Interestingly, in the CD4^+^ and CD8^+^ population, it was observed that proliferating CD4^+^ and CD8^+^ T cells failed to upregulate markers consistent with activation by an antigen specific stimulus such as CD25 and PD1 in CR group (Figure 6G–I). All immune cells evaluated decreased with time, although there was variation among the different cells with respect to when they reached peak value. NK cell populations decreased in tumor-bearing mice even after controlling for tumor burden. The population of CD4^+^PD1^+^ and CD8^+^PD1^+^ were demonstrably lower in CR mice compared with PR mice, though percentage of them respectively were similar on day 60 (Figure 6G,H). CD4^+^ Treg cell content was significantly 2-fold lower in CR mice compared with PR mice (Figure 6I). The number of MDSCs increased over time in PR animals, and then decreased slower than in CR mice. It was also observed that the number of cells were directly related to the size of the tumor (Figure 1B, Figure 5 and Figure 6).

### 2.4. Down-Regulation of Suppressor Cells in Tumor-Free Mice Rechallenged with B16F10 Cells after pIL-12 GET 

**Figure 7:**
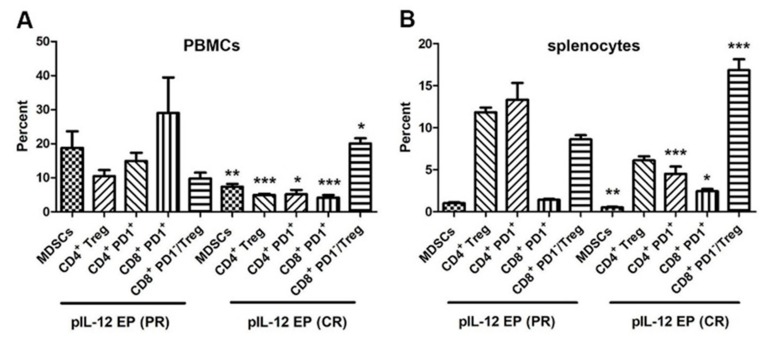
pIL-12 GET induced prevention against rechallenge via down regulation of immune suppressor cells and exhausted cells. Peripheral blood mononuclear cells (PBMCs) (**A**) and splenocytes (**B**) were harvested at 20–30 days post rechallenge with 5 × 10^5^ B16F10 cells from PR and CR mice for flow cytometry assay. Pooled data from two independent experiments are shown as mean +/− SEM (animals in each group, *n* = 6–10). Independent *T*-test, *p* * < 0.05, *p* ** < 0.01, *p* ***< 0.001.

Mice that were tumor-free for at least 50 days were rechallenged by injecting B16F10 cells into the right flank and followed for appearance of new tumor growth. A proportion of pIL-12 EP1 and pIL-12 EP2 mice were observed to maintain long-term tumor free survival (CR), or had early tumor control followed by progressive outgrowth (PR). To determine the mechanism, mice that achieved either CR or PR were compared. Previous experiments indicated that a tumor mass would form in control group within 7 days following B16F10 cells injection. For the long-term surviving mice, the tumor development was postponed until day 14 in PR group. The CR mice achieved complete prevention against rechallenge and no tumor recurrence was observed in mice [9]. Therefore, in the present study, mice that were tumor-free 20 days after rechallenge were deemed to have mounted a long-term response and were evaluated at that time point. Compared with the immune subsets changes in PR group, there were significant differences in the percentage of suppressor cells, such as MDSCs, CD4^+^ Treg, CD4^+^PD1^+^ and CD8^+^PD1^+^ from CR mice in blood circulation and spleen (Figure 7A,B and Figure S4). The down regulation of MDSCs percent was similar in the blood circulation and spleen. MDSCs are a heterogeneous population of immature myeloid cells that originate from the bone marrow [16,17]. Cytokines such as IL-12 and IFN-γ have been shown to convert MDSCs into an APC-like cell that activates and enhances the functions of T cells either in vitro [18] or in vivo [19]. The data generated in this study demonstrates that the frequency of MDSCs in blood and spleen was dramatically decreased by pIL-12 GET. Thus, MDSC-mediated immunosuppression milieu was reversed by pIL-12 GET. Given the lower percentage of CD4^+^ Treg cells, the ratio of CD8^+^PD1^−^/Treg was relatively increased, although the percentage of CD8^+^PD^−^ was not different after rechallenge.

## 3. Discussion

A major hurdle for cancer immunotherapy is that the majority of tumor cells and their associated antigens are located in an immunosuppressive tumor environment. Despite the fact that large populations of tumor-reactive T cells can be raised in patients, T cells cannot fully realize their tumoricidal potential within the tumor. Therefore, an abundance of inactivated T cells do not readily translate to tumor destruction [20]. An important component would be the conversion of the tumor microenvironment into an immunosupportive environment, thereby enabling the employment of tumor antigens in an immunostimulatory context. Immunogenic conversion of the tumor microenvironment can be achieved by intratumoral administration of appropriate immunostimulatory compounds such as IL-12. However, systemic administration of recombinant IL-12 in clinical trials for various cancers resulted in some objective responses but also resulted in severe adverse effects [21]. The goal of pIL-12 GET is to initiate or reinitiate a self-sustaining cycle of anti-tumor immunity, enabling it to amplify and propagate, but not so much as to generate unrestrained autoimmune inflammatory responses.

We and others have previously shown that local IL-12 gene therapy of malignant melanoma in patients or in preclinical models has yielded promising results with less toxicity [3]. In this study, we demonstrated that plasmid encoding mouse IL-12 (pIL-12) delivered by GET directly into the tumor was able to recruit and/or induce the proliferation of immune cells, which led to efficient tumor suppression (Figure 1B–D). Apparently, pIL-12 intratumor injection alone is insufficient to initiate an immune response cascade leading to the suppression of tumor growth. We found that through manipulation of GET parameters the onset, level, and duration of protein expression of IL-12 can be controlled, resulting in meaningful therapeutic effects in melanoma without dose-dependent IL-12 toxicities [22].

Although IL-12 has been shown to have powerful tumor-suppressing effects in a variety of animal models, the mechanistic underpinning of this phenomenon has never been fully understood, especially with respect to which immune cells are linked to the mediation of IL-12 induced tumor suppression. The data presented in this study shows that beyond the extent of infiltration, the balance and organization of individual TILs subpopulation are important parameters of B16F10 melanoma immune microenvironment. B16F10 melanoma immune infiltrate forms a non-continuum (Figure 2) where the TILs density decreased progressively from tumor periphery to tumor mass in the no TX group, EP1 and EP2. However, the location of TILs moved from the periphery to the tumor mass and the density of TILs increased after pIL-12 injection only and especially after pIL-12 GET treatment. We evaluated CD3^+^, CD4^+^ and CD8^+^ T cell density and frequency. pIL-12 GET resulted in significant expansion in total number of CD3^+^ T cells (Figure 2A). Despite the decreased-percentage of PD1 positive cells and Foxp3 positive cells in CD4^+^ and CD8^+^ T cells following treatment with EP1 or EP2 without pIL-12 (Figure 4C–F), tumor growth continued in EP1 and EP2 groups (Figure 1B). At initial evaluation, this would seem counter to our supposition that conversion from a suppressive environment to an immune inducing environment and better anti-tumor activity. However, while the percentages of these cells were reduced, the number of infiltrating CD3^+^ cells were greatly reduced compared to pIL-12 GET EP1 or pIL-12 GET EP2 or even pIL-12 injection only (Figure 2, Figure 3 and Figure 4). The key to an effective anti-tumor immune response is critically related to the robustness of the lymphocytic infiltrate as well as the reduction of Treg and PD1^+^ expression. Overall, our interpretation of this data is that increases in CD3^+^ T cells within the TME in tumor-bearing mice do impact tumor growth. Specifically, in this case, IL12 GET therapy drove an increase in effector T cells. In addition, IL-12 GET therapy resulted in a much lower percentage of Treg and PD1^+^ cells. Together these factors resulted in much more pronounced anti-tumor effect. With respect to EP1 and EP2, while there was a lower percentage of Treg and PD1^+^ cells, the overall number of CD3 cells were not sufficient to induce an effective immune response against the tumor. In line with an increase of CD3^+^ T cells in total numbers, the frequency of CD8^+^ T cells was also significantly expanded. In contrast, total CD4^+^ T cell frequency decreased compared with the ratio of CD4^+^ to CD8^+^ T cells in peripheral blood and spleen, although there was significantly higher frequency compared with no TX group. When we assessed the CD4^+^ Foxp3^+^ Treg, CD4^+^PD1^+^ and CD8^+^PD1^+^ T cells, the frequency decreased compared with no TX. Notably, pIL-12 GET recruited abundance of CD4^+^ T cells, CD8^+^ T cells and modified their function within the tumor microenvironment. The results from the TILs revealed a significantly stronger CD8^+^ T-cell tumor infiltration, a higher CD8^+^/CD4^+^ T-cell ratio, and higher CD8^+^ PD1^−^/Treg-cell ratio in the group of pIL12 GET. High ratios between CD8^+^ T cells and the other cell types were associated with improved survival [14,23,24]. In the present study, PD1 expression was down-regulated after pIL12 GET. These results were in line with other reports [25,26]. Although reports in the literature regarding the regulation of PD1 expression by IL12 are conflicting, we showed that the percentage of PD1^+^ T cells decreased in EP1 and EP2 (Figure 4F) and intratumor pIL12 GET reduced the percentage of CD8^+^PD1^+^ in CD8^+^ T cells. Since the number of lymphocytes impact the therapeutic efficacy of immnunotherapy and pIL12 GET increases lymphocytic infiltrate into the tumor microenvironment as well as leads to reduction of PD1 expression, it is clear that this approach can play an important role in enhanced antitumor immunotherapy through the expansion of effector T cells infiltrating into the tumor. However, the mechanism behind these phenomena still needs further exploration to be completely delineated.

We propose that peripheral blood monitoring can be used to study responses to pIL-12 GET induced protection from tumor challenge. Previous studies have shown that IL-12 mediated anti-tumor immune response was due to T cells and NK cells [4,5,27], so we assessed T and NK cell phenotypes in peripheral blood. Proliferation of memory (CD44^+^) CD4^+^ and CD8^+^ T cells are the major component for cellular immunotherapy, therefore we also evaluated if there were differences in CD4^+^ and CD8^+^ T memory phenotype across the experimental timeframe. Here, we showed that pIL-12 GET is a strong immunostimulatory therapy which generally induced CD4^+^ and CD8^+^ T cells expansion for cancer. In addition, treatment with pIL-12 GET can induce a potent proliferation of memory (CD44^+^) CD4^+^ and CD8^+^ T cells, but not NK cells in the peripheral blood (Figure 5 and Figure 6) leading to induction of protection from tumor relapse and metastasis. The results also indicated that the immune response induced a down-regulation of suppressor cells in mice rechallenged with B16F10 cells after pIL-12 GET (Figure 7). To confirm these results, future experiments will explore depletion of specific immune subsets, such as CD4^+^, CD8^+^ T cells, NK and Gr-1^+^ cells.

There are multiple studies that have provided evidence that breaking the stronghold of MDSCs in the tumor microenvironment is a key step toward effective antitumor immunity. IL-12 and IFN-γ have the capacity to convert MDSCs into functional, nonsuppressive antigen-presenting cells [19,27,28]. Our previous studies have shown that treatment with pIL-12 GET therapies for cancer, results in preferential induction of IFN-γ production [9]. IL-12 led to higher expression of IFN-γ within the tumor microenvironment, which has been linked to tumor control. Consistent with these previous studies, we showed that pIL-12 GET treatment decreased in vivo MDSC infiltration of tumors and increased CD8^+^ T cell infiltration and survival. The mechanism by which pIL-12 GET alters the tumor microenvironment revealed that IL-12 initiated infiltration of immune cells into tumor mass, which in turn changes the tumor microenvironment from a suppressive condition to an antitumor milieu. Given that PDL1 expression in B16F10 cells (Figure S2) and PD1 expression in CD8^+^ T cells (Figures S3 and S4) and that IL-12 activates a local immune response in the primary melanoma via the recruitment of T lymphocytes to the tumor site as well as triggering the exposure and recognition of tumor antigens, it may be possible to enhance the antitumor effect by combining pIL-12 GET with checkpoint inhibitors. This possibility is now being explored, with plans to combine IL-12-mediated local tumor suppression with blockade of co-inhibitory molecules against PD1 on T cells or PDL1 on B16F10 melanoma tumor cells.

Although the reasons for the limited clinical efficacy of IL-12 in cancer patients is not fully understood [8,15,28], several immunosuppressive mechanisms, including CD4^+^ Treg cell accumulation due to increased IL-10 production and diminished IFN-*γ* production after repetitive treatments with IL-12, could be involved [17,29,30]. In addition, immune suppressive microenvironment characterized by infiltration of MDSCs in advanced tumors [16,31] could also contribute to the limited efficacy (Figure S3). To improve the therapeutic efficacy with IL-12 but simultaneously minimize the toxicity, the next plan will include targeting of pIL-12 to only tumor, and co-administration with a plasmid encoding anti-CD25 plasmid in order to deplete Treg cells or a plasmid encoding anti-Ly6G to deplete MDSCs. Thus, IL-12 has a high potential to be used successfully for cancer immunotherapy.

## 4. Materials and Methods

### 4.1. Cell Lines, Mice and Tumor Formation

B16F10 murine melanoma cells (ATCC) were maintained in Dulbecco’s Modified Eagle Medium (DMEM) supplemented with 10% FCS and 0.2% gentamicin. All mouse studies were performed with the approval of the Old Dominion University Institutional Animal Care and Use Committee. Six-eight-week-old female C57BL/6J mice were obtained from Charles River Laboratories (Wilmington, MA, USA) and maintained in a specific pathogen-free condition in the host laboratory. The left flank of female C57BL/6J mice was shaved and 1 × 10^6^ cells in 50 µL of sterile 0.9% saline were injected subcutaneously. Tumors were measured using digital calipers, and treatment was begun when tumors reached 50–70 mm^3^ in volume. Treatment was performed on Days 0, 4 and 7. Intratumoral injection of plasmid mouse IL-12 (pIL-12, 50 μg/50 μL) with EP1 (six 1300 V/cm, 100 μs pulses with an array of 6 penetrating electrodes (circular 1 cm diameter array), pIL-12 EP1) or EP2 (ten 600 V/cm, 5 ms pulses with a non-penetrating caliper electrodes, pIL-12 EP2). Tumor volume was calculated using the formula v = 0.52 × length × width^2^. For rechallenge, mice were injected with 5 × 10^5^ B16F10 cells in the right flank.

### 4.2. Plasmid DNA

pUCMV3-mIL-12 (pIL-12, Aldevron, Fargo, ND, USA), containing the murine p35 and p40 IL-12 cDNAs under the control of the cytomegalovirus promoter, was prepared by the manufacturer [3].

### 4.3. Flow Cytometry for Membrane Staining

EDTA anticoagulant whole blood was used with conjugated antibodies as previously described [12]. After addition of conjugated antibodies, Optilyse B Lysing Solution (Beckman Coulter, Pasadena, CA, USA) was used to lyse blood cells. Single spleen cells were mixed with antibodies according the protocols, after enterocytes lysing with ASK (A1049201, ThermoFisher Scientific, Waltham, MA, USA). TILs were isolated from tumor tissues by Ficoll-Paque density gradient. Stained-TILs were evaluated using flow cytometry MACSquant analyzer 10 (Miltenyi, Bergisch Gladbach, Germany).

The following mAbs were used: PE vio770-conjugated anti-CD3 (REA 641), vioblue-conjugated anti-CD4 (REA 604), APC-vio770-conjugated anti-CD8a (53-6.7), APC-conjugated anti-CD25 (REA 568), FITC-conjugated anti-CD127 (A7R34), PE-conjugated anti-PD1 (REA 802), PE-conjugated anti-NK1.1 (PK136), APC-conjugated anti-CD11b (REA 596), vioblue-conjugated anti-Gr-1 (RB6-8C5), PerCP vio 700-conjugated anti-CD45 (REA737), FITC-conjugated anti-CD44 (REA 664), APC-conjugated anti-CD62L (REA 828). The above antibodies were purchased from Miltenyi (Miltenyi, Bergisch Gladbach, Germany). FITC-conjugated anti--NKp46 (29A1.4) was purchased from Bio Legend (San Diego, CA, USA), and analyzed using MACSquant analyzer 10. Additional analysis was performed using FlowJo software V10 (Tree Star, Inc., Ashland, OR, USA).

### 4.4. Immunohistochemistry

Fixed-tumor tissues were excised from tumor-bearing mice and fixed using IHC Zinc Fixative (BD Bioscience, San Jose, CA, USA). Five micrometer paraffin-embedded sections were prepared for Immunohistochemical staining with Tyramide Signal Amplification (Opal Multiplex IHC Assay, PerkinElmer, Waltham, MA, USA) to examine the percentage of TILs. The following primary antibodies were used: rat anti-mouse CD3 (CD3-12), rabbit anti-mouse CD4 (EPR19514), rat anti-mouse CD8a (4SM15), rat anti-mouse Foxp3 (FJK-16S), rabbit anti-mouse PD1 (EPR20665). The number of immune cells per section was recorded blind to genotype and normalized to core area. Individual core counts from 10 or more replicates were available for most cases, and immune cell counts per square millimeter were averaged across replicates. Cut-off values of low versus high immune cells were defined by the midpoint. Slides were examined with an Olympus BX51 microscope using Camera Software for DP80 (Olympus America Inc., Center Valley, PA, USA). The cell count of TILs was determined using ImageJ. The density of TILs calculation as follows:

$$density\;of\;TIL=\frac{cells\;count\;number}{size\;of\;image\;field\;of\;view\;(mm^{2})}$$

$$\begin{array}{ll} image\;field\;of & \;view\;\left(height,\;width,\;diagonal\right)\\ & =\frac{\mathrm{CCD}\;\mathrm{sensor}\;\mathrm{size}\;\left(\mathrm{height},\;\mathrm{width},\;\mathrm{diagonal}\right)}{\mathrm{objective}\;\mathrm{magnification}\;x\;\mathrm{adapter}\;\mathrm{magnification}} \end{array}$$

### 4.5. Granzyme B ELISPOT

B16F10 tumor-bearing mice were euthanized and spleens removed. Effector cells from splenocytes or the positive activation control ConA at 5 mg/mL (Sigma-Aldrich, St Louis, MO, USA) were incubated with B16F10 target cells for 48 h in 96-well plates in triplicate [12]. Granzyme B enzyme-linked immune spot (ELISPOT) assays were performed according to manufacturer’s protocol using a commercial Mouse Granzyme B ELISPOT kit (R&D system, Minneapolis, MN, USA). The plates were scanned and analyzed on an ImmunoSpot ELISPOT Reader (Cellular Technology Limited, Clevland, OH, USA).

### 4.6. Cell-Killing Assay with Flow Cytometry

For the killing assay [12], B16F10 tumor cells were labeled with 1 µM carboxyfluorescein succinimidyl ester (CFSE, Biolegend, San Diego, CA, USA) and used as targets. Effecter cells from spleen were cultured in round-bottom polystyrene tubes with CFSE labeled target B16F10 cells. After 4 h of incubation of target and effector cells, propidium iodide (PI) (1 μg/mL, Sigma–Aldrich) was added. The cytotoxic activity was measured by flow cytometry analysis comparing CFSE^+^PI^+^cells (killed targets) with CFSE^+^PI^−^cells (vital targets). Approximately 5000 target cells were acquired.

### 4.7. Statistical Analysis

Data were expressed as the mean ± standard error of the mean (SEM). For differences between groups, one-way ANOVA was used for three or more groups, and student’s *t*-test was used for two groups. Survival data from the animal studies were analyzed using the log-rank test. Statistical analyses were performed with commercially available software (SPSS 16.0 and GraphPad Prism 5, San Diego, CA, USA).

## 5. Conclusions

In summary, we show that pIL-12 GET alters the tumor microenvironment from suppressive condition to antitumor milieu. These data highlight that subsets of CD44^+^ T memory cells in circulation are representative of cells at immune sites and underscore the importance of evaluating peripheral blood when making determinations about immune surveillance being able to successfully prevent tumor relapse and metastasis. More broadly speaking, these findings may guide the development of combination cancer therapies to make tumors more accessible for targeted immune therapy and vaccination to establish long-term antitumor immunity in patients.

## 6. Patents

Richard Heller is an inventor on patents, which cover the technology that was used in the work reported in this manuscript. 

## Supplementary Materials

The following are available online at http://www.mdpi.com/2072-6694/10/12/498/s1, Figure S1. pIL-12 GET therapy associated with minimal systemic toxicity; Figure S2. H-2Kb and PDL1 expression in B16F10 melanoma tumor cells; Figure S3. Exhausted CD8+PD1-, CD4+ Treg in tumor-infiltrating lymphocytes (TILs); Figure S4. The changes of immune cells in pIL-12 GET induced prevention of new tumor formation following rechallenge.

## References

[1] Maverakis E., Cornelius L.A., Bowen G.M., Phan T., Patel F.B., Fitzmaurice S., He Y., Burrall B., Duong C., Kloxin A.M. (2015). Metastatic melanoma—A review of current and future treatment options. Acta Dermato-Venereologica.

[2] Lui P., Cashin R., Machado M., Hemels M., Corey-Lisle P.K., Einarson T.R. (2007). Treatments for metastatic melanoma: Synthesis of evidence from randomized trials. Cancer Treat. Rev..

[3] Heller L., Merkler K., Westover J., Cruz Y.D., Coppola K., Benson A., Daud R. (2006). Heller, Evaluation of toxicity following electrically mediated interleukin-12 gene delivery in a B16 mouse melanoma model. Clin. Cancer Res..

[4] Brunda M.J., Luistro L., Warrier R.R., Wright R.B., Hubbard B.R., Murphy M., Wolf S.F., Gately M.K. (1993). Antitumor and antimetastatic activity of interleukin 12 against murine tumors. J. Exp. Med..

[5] Smyth M.J., Taniguchi M., Street S.E. (2000). The anti-tumor activity of IL-12: Mechanisms of innate immunity that are model and dose dependent. J. Immun..

[6] Motzer R.J., Rakhit A., Schwartz L.H., Olencki T., Malone T.M., Sandstrom K., Nadeau R., Parmar H., Bukowski R. (1998). Phase I trial of subcutaneous recombinant human interleukin-12 in patients with advanced renal cell carcinoma. Clin. Cancer Res..

[7] Bortolanza S., Bunuales M., Otano I., Gonzalez-Aseguinolaza G., Ortiz-de-Solorzano C., Perez D., Prieto J., Hernandez-Alcoceba R. (2009). Treatment of pancreatic cancer with an oncolytic adenovirus expressing interleukin-12 in Syrian hamsters. Mol. Ther..

[8] Daud A.I., DeConti R.C., Andrews S., Urbas P., Riker A.I., Sondak V.K., Munster P.N., Sullivan D.M., Ugen K.E., Messina J.L. (2008). Phase I trial of interleukin-12 plasmid electroporation in patients with metastatic melanoma. J. Clin. Oncol..

[9] Lucas M.L., Heller L., Coppola D., Heller R. (2002). IL-12 plasmid delivery by in vivo electroporation for the successful treatment of established subcutaneous B16.F10 melanoma. Mol. Ther..

[10] Lucas M.L., Heller R. (2003). IL-12 gene therapy using an electrically mediated nonviral approach reduces metastatic growth of melanoma. DNA Cell Biol..

[11] Chandran S.S., Somerville R.P.T., Yang J.C., Sherry R.M., Klebanoff C.A., Goff S.L., Wunderlich J.R., Danforth D.N., Zlott D., Paria B.C. (2017). Treatment of metastatic uveal melanoma with adoptive transfer of tumour-infiltrating lymphocytes: A single-centre, two-stage, single-arm, phase 2 study. Lancet. Oncol..

[12] Shi G., Zhou C., Wang D., Ma W., Liu B., Zhang S. (2014). Antitumor enhancement by adoptive transfer of tumor antigen primed, inactivated MHC-haploidentical lymphocytes. Cancer Lett..

[13] Tietze J.K., Wilkins D.E., Sckisel G.D., Bouchlaka M.N., Alderson K.L., Weiss J.M., Ames E., Bruhn K.W., Craft N., Wiltrout R.H. (2012). Delineation of antigen-specific and antigen-nonspecific CD8(+) memory T-cell responses after cytokine-based cancer immunotherapy. Blood.

[14] Piersma S.J., Jordanova E.S., van Poelgeest M.I., Kwappenberg K.M., van der Hulst J.M., Drijfhout J.W., Melief C.J., Kenter G.G., Fleuren G.J., Offringa R. (2007). High number of intraepithelial CD8+ tumor-infiltrating lymphocytes is associated with the absence of lymph node metastases in patients with large early-stage cervical cancer. Cancer Res..

[15] Car B.D., Eng V.M., Lipman J.M., Anderson T.D. (1999). The toxicology of interleukin-12: A review. Toxicol. Pathol..

[16] Gabrilovich D.I., Nagaraj S. (2009). Myeloid-derived suppressor cells as regulators of the immune system. Nat. Rev. Immunol..

[17] Haicheur N., Escudier B., Dorval T., Negrier S., De Mulder P.H., Dupuy J.M., Novick D., Guillot T., Wolf S., Pouillart P. (2000). Cytokines and soluble cytokine receptor induction after IL-12 administration in cancer patients. Clin. Exp. Immunol..

[18] Bronte V., Apolloni E., Cabrelle A., Ronca R., Serafini P., Zamboni P., Restifo N.P., Zanovello P. (2000). Identification of a CD11b(+)/Gr-1(+)/CD31(+) myeloid progenitor capable of activating or suppressing CD8(+) T cells. Blood.

[19] Kerkar S.P., Goldszmid R.S., Muranski P., Chinnasamy D., Yu Z., Reger R.N., Leonardi A.J., Morgan R.A., Wang E., Marincola F.M. (2011). IL-12 triggers a programmatic change in dysfunctional myeloid-derived cells within mouse tumors. J. Clin. Invest..

[20] Mocellin S., Rossi C.R., Nitti D. (2004). Cancer vaccine development: On the way to break immune tolerance to malignant cells. Exp. Cell Res..

[21] Cavallo F., Di Carlo E., Butera M., Verrua R., Colombo M.P., Musiani P., Forni G. (1999). Immune events associated with the cure of established tumors and spontaneous metastases by local and systemic interleukin 12. Cancer Res..

[22] Shirley S.A., Lundberg C.G., Li F., Burcus N., Heller R. (2015). Controlled gene delivery can enhance therapeutic outcome for cancer immune therapy for melanoma. Curr. Gene Ther..

[23] Sato E., Olson S.H., Ahn J., Bundy B., Nishikawa H., Qian F., Jungbluth A.A., Frosina D., Gnjatic S., Ambrosone C. (2005). Intraepithelial CD8+ tumor-infiltrating lymphocytes and a high CD8+/regulatory T cell ratio are associated with favorable prognosis in ovarian cancer. PNAS.

[24] Diederichsen A.C., Hjelmborg J., Christensen P.B., Zeuthen J., Fenger C. (2003). Prognostic value of the CD4+/CD8+ ratio of tumour infiltrating lymphocytes in colorectal cancer and HLA-DR expression on tumour cells. Cancer Immunol. Immunother..

[25] Ngiow S.F., Young A., Blake S.J., Hill G.R., Yagita H., Teng M.W., Korman A.J., Smyth M.J. (2016). Agonistic CD40 mAb-Driven IL12 Reverses Resistance to Anti-PD1 in a T-cell-Rich Tumor. Cancer Res..

[26] Gerner M.Y., Heltemes-Harris L.M., Fife B.T., Mescher M.F. (2013). Cutting edge: IL-12 and type I IFN differentially program CD8 T cells for programmed death 1 re-expression levels and tumor control. J. Immunol..

[27] Steding C.E., Wu S.T., Zhang Y., Jeng M.H., Elzey B.D., Kao C. (2011). The role of interleukin-12 on modulating myeloid-derived suppressor cells, increasing overall survival and reducing metastasis. Immunology.

[28] Ryffel B. (1997). Interleukin-12: Role of interferon-gamma in IL-12 adverse effects. Clin. Immunol. Immunopath..

[29] Beyer M., Schultze J.L. (2006). Regulatory T cells in cancer. Blood.

[30] Portielje J.E., Lamers C.H., Kruit W.H., Sparreboom A., Bolhuis R.L., Stoter G., Huber C., Gratama J.W. (2003). Repeated administrations of interleukin (IL)-12 are associated with persistently elevated plasma levels of IL-10 and declining IFN-gamma, tumor necrosis factor-alpha, IL-6, and IL-8 responses. Clin. Cancer Res..

[31] Mocellin S., Rossi C.R., Pilati P., Nitti D. (2005). Tumor necrosis factor, cancer and anticancer therapy. Cytokine Growth Factor Rev..

