# Supplementary material for: IL-12 Gene Electrotransfer Triggers a Change in Immune Response within Mouse Tumors
Source: Cancers (Basel). 2018 Dec 8;10(12):498. doi: 10.3390/cancers10120498 (PMC6315808; doi:10.3390/cancers10120498)
Supplement: Supplementary file 1 [file cancers-10-00498-s001.pdf]

Supplementary Materials: IL-12 Gene Electrotransfer Triggers a Change in Immune Response within Mouse Tumors

Guilan Shi, Chelsea Edelblute, Sezgi Arpag, Cathryn Lundberg and Richard Heller

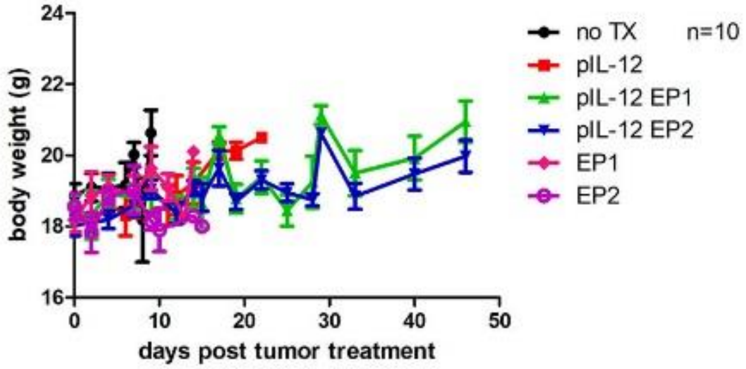

Figure S1. pIL-12 GET therapy associated with minimal systemic toxicity. On day 7, C57BL/6 mice were inoculated with B16F10 cells (1 × 10⁶/50µL, s.c in the left flank.). Tumor-bearing C57BL/6 mice were treated with pIL-12 GET on day 0, 4 and 7. Animal weight data. Tumor-bearing mice did not show weight loss comparing the no TX group. The data presented are representative of two independent experiments. Each value represents the mean +/− SEM of the group (animals in each group, *n* = 8~13).

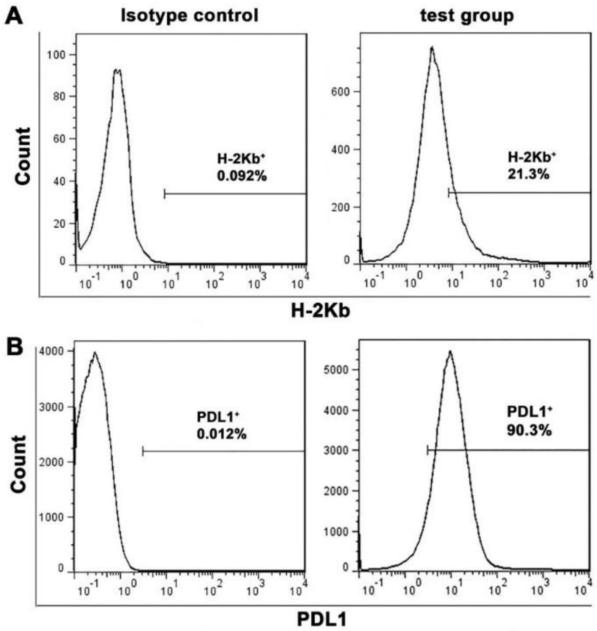

Figure S2. H-2Kb and PDL1 expression in B16F10 melanoma tumor cells. The H-2Kb and PDL1 expression in B16F10 tumor cells from tumor tissue (no TX group) were detected with flow cytometry at day 9. H-2Kb (**A**) and PDL1 (**B**) expression in B16F10 melanoma cells.

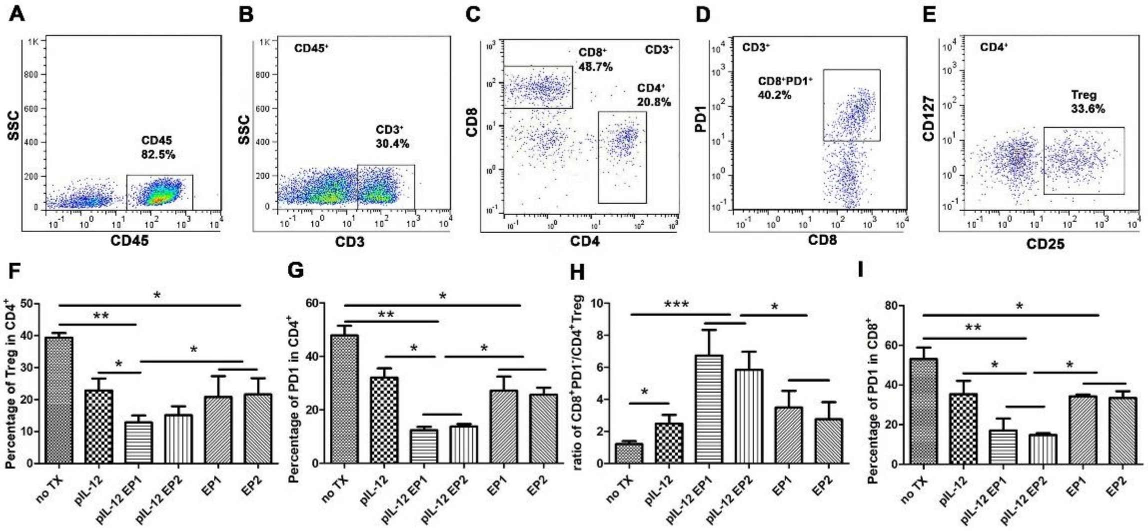

Figure S3. Exhausted CD8⁺PD1⁻, CD4⁺ Treg in tumor-infiltrating lymphocytes (TILs). TILs from tumors tissue were collected at day 9 for flow cytometry assay. (**A**–**E**) Flow cytometry gating strategy used for defining immune cell subsets. (**F**–**I**) CD4⁺ Treg, CD4⁺PD1⁺, ratio of CD8⁺PD1⁻/Treg, Exhausted CD8⁺PD1⁺ in TILs. One-way ANOVA, p * < 0.05, p ** < 0.01, p ***< 0.001.

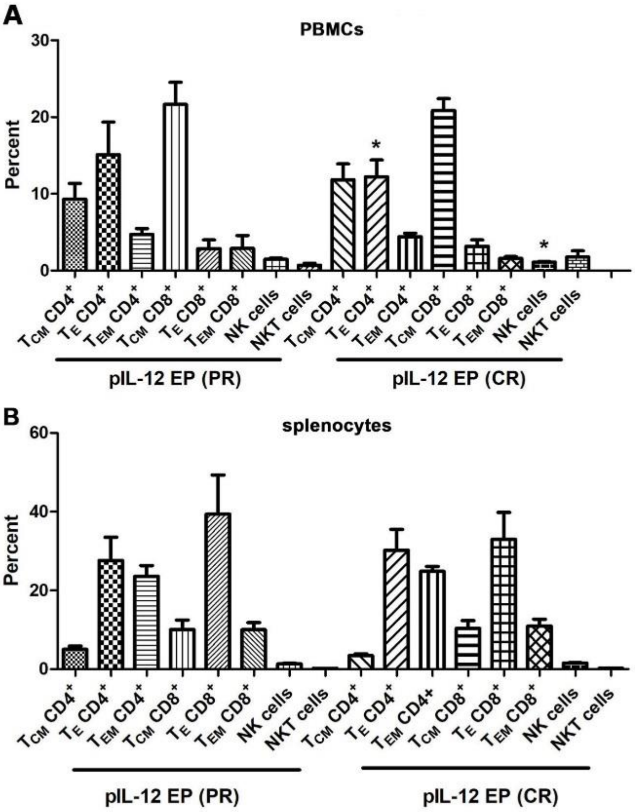

Figure S4. The changes of immune cells in pIL-12 GET induced prevention of new tumor formation following rechallenge. Peripheral blood mononuclear cells (PBMCs) (**A**) and splenocytes (**B**) were harvested at 20~30 days post rechallenge with 5 × 10^5 B16F10 cells from PR and CR mice for flow cytometry assay. Pooled data from two independent experiments are shown as mean +/− SEM. (animals in each group, n = 8~13). Independent t-test, p * < 0.05.
